# Correction: Is Chronic Suppurative Otitis Media a Neglected Tropical Disease?

**DOI:** 10.1371/journal.pntd.0003761

**Published:** 2015-04-27

**Authors:** The PLOS Neglected Tropical Diseases Staff

## Notice of Republication

This article was republished on April 10, 2015 to fix errors with the reference links within the article that were introduced at copyedit. The publisher apologizes for the errors. Please download this article again to view the correct version. The originally published, uncorrected article and the republished, corrected article are provided here for reference.

## Supporting Information

S1 File Originally published, uncorrected article. (PDF)

S2 File Republished corrected article. (PDF)
