# Supplementary material for: Correction: Is Chronic Suppurative Otitis Media a Neglected Tropical Disease?
Source: PLoS Negl Trop Dis. 2015 Apr 27;9(4):e0003761. doi: 10.1371/journal.pntd.0003761 (PMC4410952; doi:10.1371/journal.pntd.0003761)
Supplement: S1 File — (PDF) [file pntd.0003761.s001.pdf]

PLOS | NEGLECTED TROPICAL DISEASES

EDITORIAL

Is Chronic Suppurative Otitis Media a Neglected Tropical Disease?

Michael G. Li[1]*, Peter J. Hotez[1,2,3,4], Jeffrey T. Vrabec[5], Donald T. Donovan[5]*

1 Baylor College of Medicine, School of Medicine and National School of Tropical Medicine, Houston, Texas, United States of America, **2** Sabin Vaccine Institute and Texas Children's Hospital Center for Vaccine Development, Houston, Texas, United States of America, **3** James A Baker III Institute for Public Policy, Rice University, Houston, Texas, United States of America, **4** Department of Biology, Baylor University, Waco, Texas, United States of America, **5** Baylor College of Medicine, Department of Otolaryngology-Head and Neck Surgery, Houston, Texas, United States of America

* Michael.Li@bcm.edu (MGL); ddonovan@bcm.edu (DTD)

The World Health Organization has identified chronic suppurative otitis media (CSOM) in an expanded list of neglected conditions (beyond its list of 17 neglected tropical diseases). We address CSOM's potential status as a true neglected tropical disease given its prevalence across the indigent populations of developing countries and its influence both from a medical and socioeconomic perspective.

CrossMark
click for updates

OPEN ACCESS

Citation: Li MG, Hotez PJ, Vrabec JT, Donovan DT (2015) Is Chronic Suppurative Otitis Media a Neglected Tropical Disease? PLoS Negl Trop Dis 9 (3): e0003485. doi:10.1371/journal.pntd.0003485

Editor: Melinda Mary Pettigrew, Yale University, UNITED STATES

Published: March 26, 2015

Copyright: © 2015 Li et al. This is an open access article distributed under the terms of the Creative Commons Attribution License, which permits unrestricted use, distribution, and reproduction in any medium, provided the original author and source are credited.

Funding: There was no specific funding for this article.

Competing Interests: The authors have declared that no competing interests exist.

Chronic suppurative otitis media (CSOM) is one of the most common childhood diseases worldwide, and according to the World Health Organization (WHO), it affects anywhere between 65 and 330 million people worldwide, but mainly in the developing countries [1,2,3]. A recent epidemiological study estimated that countries with the highest incidence rates of CSOM resided in impoverished tropical and subtropical regions, although there is also an abnormally high prevalence of CSOM among vulnerable populations in high-income countries such as Aboriginal and indigenous peoples living in Australia and Alaska, United States, respectively [2,4] (Fig. 1). Although the exact definition of CSOM is still under debate, WHO currently defines it as a chronic inflammation of the middle ear cavity with recurrent discharge through tympanic perforation for a period of three months or greater [2] (Fig. 2). CSOM is believed to occur most frequently within the first six years of childhood, often following poor management of acute otitis media (AOM) [2,5,6]. Because the exact point in time as to when AOM becomes CSOM is still heavily debated, accurate diagnosis of CSOM remains a difficult task. Overall, the Global Burden of Disease Study 2010 assigned 4.68 million disability-adjusted life years (DALYs) to otitis media, a disease burden that is almost as high as the intestinal helminth infections [7].

Complicating factors for CSOM include an obstructed view of the tympanic membrane by otorrhea and unreliable patient history regarding the frequency and duration of otitis media episodes [2]. These clinical obstacles, along with the high prevalence in developing countries, have led WHO to recognize CSOM in an expanded list of neglected conditions beyond its group of 17 neglected tropical diseases (NTDs) [8]. However, there are many additional complications and sequelae. Extracranial complications like mastoid abscess and facial nerve palsy, as well as life-threatening intracranial complications like brain abscess and meningitis have been associated with CSOM [1,2,9]. These findings, along with studies showing CSOM as a leading cause of mild to moderate conductive hearing loss, suggest that CSOM could be considered as a true NTD [1,2].

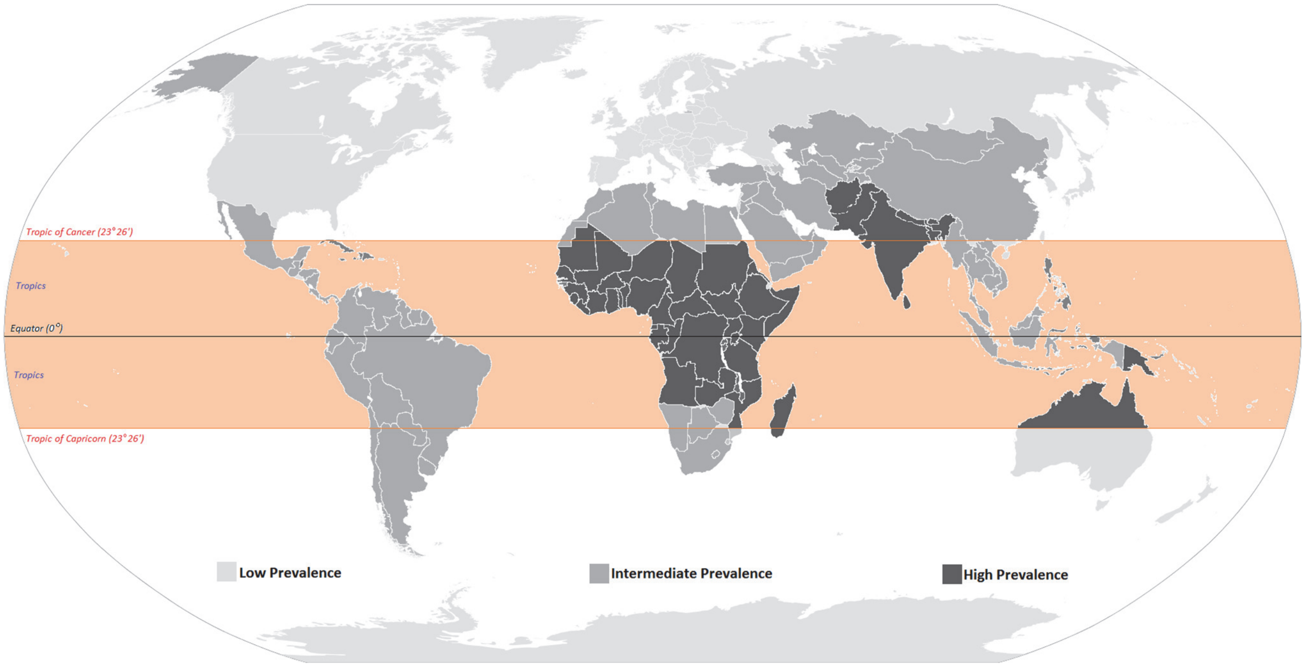

Fig 1. Prevalence of CSOM worldwide. Figure modified from Wikipedia BlankMap-World6.svg, available here: http://en.wikipedia.org/wiki/File:BlankMap-World6.svg; and from Figure 4 and data in Monasta et al., reference 4.

doi:10.1371/journal.pntd.0003485.g001

Most NTDs also have significant socioeconomic consequences. CSOM is no different. Compounding the adverse health effects of CSOM are its widespread and significant developmental and social implications.

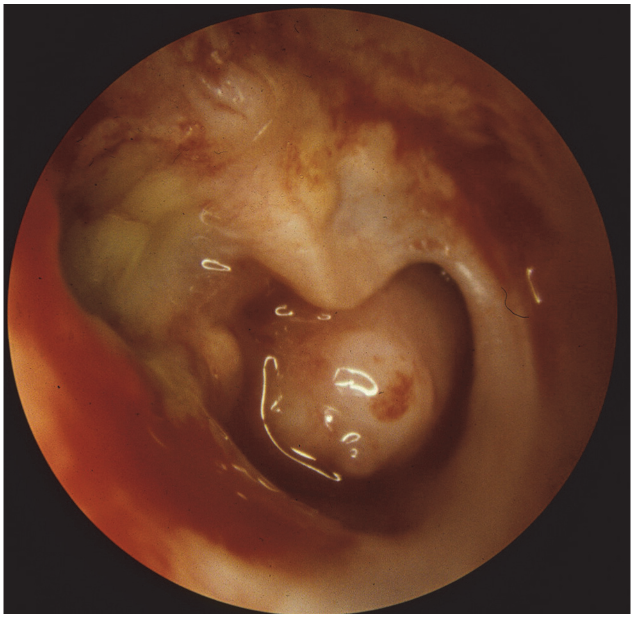

Fig 2. Right ear showing subtotal perforation, incus erosion, and chronic inflammation of the middle ear mucosa with mucoid drainage. Image courtesy of Dr. Jeffrey T. Vrabec.

doi:10.1371/journal.pntd.0003485.g002

The relationship between hearing loss and academic achievement has been well documented in children. Since moderate conductive hearing loss is one of the major complications associated with CSOM, it is important to measure the intellectual development of children who are afflicted with CSOM. Schoolchildren of different socioeconomic class in Yemen and Nigeria with CSOM and hearing loss have demonstrated a statistically significant drop in their scholastic performance, in which test scores and speech development all lagged behind those in healthier children [5,6,10]. Based on studies from Erick Hanushek and Paul Peterson at the Hoover Institution, which link reduced academic performance to wage-earning and economic prosperity, it is reasonable to conclude that CSOM may have a significant economic impact on the world's developing countries with people who live in extreme poverty [11].

It is also important to address the social drawbacks that CSOM patients face in addition to their intellectual development. More than 70% of CSOM patients in Nigeria report significant social embarrassment resulting from recurrent ear discharges and hearing loss [1]. However, it is interesting to note that otorrhea is often considered to be a "normal" part of childhood in developing countries where there are high rates of CSOM, and patients often do not even bring up these problems in their history [2]. Studies have shown that more than 50% of parents with children with otitis media in developing countries have deficits in knowledge of this disease [12]. This lack of understanding unfortunately is one of the reasons why CSOM continues to be overlooked and underdiagnosed. Therefore, raising awareness and educating the public is crucial, as was the case for diseases such as tuberculosis, malaria, and other NTDs. Furthermore, while there have been no studies investigating depression as a complication of CSOM, it has already been established that there is a strong association between hearing impairment and depression in adults [13].

WHO's recognition of the high prevalence of CSOM in impoverished countries and the debilitating challenges the disease presents among children living in poverty is an important first step in elevating its importance as a global health threat. Among the measures needed to prevent CSOM and its complications is a four step initiative known as HEAR—Hygiene of the ear, Early management of AOM, Antibiotics, and Raising awareness [8]. Hygiene is especially challenging given the poor standards of living in some developing countries. As a result, it is common to see children with persistent rhinorrhea and recurrent upper respiratory tract infections (URI). Many studies have demonstrated a causal relationship between recurrent URIs and otitis media [14,15,16,17]. Thus identifying the causes of the URIs and persistent rhinorrhea (e.g., enlarged adenoids, allergy, and sinusitis) may play an important role in the prevention of otitis media and ultimately CSOM. Towards the end of 2009, WHO conducted a systematic analysis of the impact of shortened ($\leq$3 days) courses of antibiotics (including azithromycin) for treating acute otitis media and as a form of preventive chemotherapy for CSOM [18]. Amoxicillin has also been shown to reduce the number of tympanic membrane perforations and slow the progression of otitis media with effusion in Aboriginal infants, suggesting a possible protective measure against CSOM [19]. However, the cost of production and delivery of these antibiotics to developing countries may still be a challenge [20].

Long standing disease may result in chronic tympanic membrane perforations and cholesteatoma formation. Treatment of these complications in underdeveloped countries will present different challenges, thus methods employed in the US are not necessarily applicable. The obstacles for treatment of anatomical consequences of otitis media include training of physicians, procurement of specialized instrumentation including operating microscopes, and adequate hospital facilities and support services. Patient factors are proximity to the treatment facility and ease of transportation. Even when these logistical hurdles are cleared, reported outcomes of surgical management by medical missions remain poorer than in developed countries [21].

The reasons for poorer outcomes include inadequate follow up care or, most likely, high disease recurrence rate.

Hearing aids are employed in developed countries for conductive hearing losses. The application of this technology in an underdeveloped region is not likely to succeed if the infrastructure for powering the device and performing proper maintenance is not in place. Currently, there is urgency to push for the development of affordable and high quality hearing aids, such as the efforts by the nongovernmental organization (NGO), World Wide Hearing [22]. However, accomplishing such a task will require the cooperation of multiple stakeholders beyond NGOs, including philanthropists, manufacturers, and member states [22]. Of course, engaging the governments of countries with high CSOM prevalence to develop prevention and management programs that target the disease in a timely manner would also be crucial in reducing disease burdens.

The cost of surgery, antibiotics, and hearing aids is high, while their impact on the underlying disease incidence is nearly negligible. As a result, it is necessary to come up with a solution that can fight CSOM in a rapid and more cost efficient manner. Vaccines against the bacterial causes of CSOM could prove to be an effective solution with results similar to the ones seen with the rapid impact package treatment of helminth infections. Prevention by vaccination is a well-supported explanation for the decline of chronic ear diseases in the more developed parts of the world [2]. Current investigation on the use of vaccines against CSOM in developing countries is heavily lacking. One study has investigated the efficacy of treating CSOM with Broncasma Berna, an inactivated bacterial vaccine [23]. These patients (median age: 57 years) with CSOM were given subcutaneous injections of the vaccine and showed a marked decrease in the amount of otorrhea with tympanic membranes that were almost completely dry within two months. A more recent study investigated the efficacy of pneumococcal vaccination in Aboriginal children in Australia for the prevention of otitis media [24]. Although there were no significant changes in the prevalence or onset of AOM, vaccinated subjects appeared to experience a reduced recurrence of tympanic membrane perforation. Studies have also shown an association between the nasopharyngeal colonization of *Staphylococcus pneumoniae* and AOM, thus searching for a vaccine that targets all relevant serotypes may provide a method to halt the development of CSOM early on [25]. Although these results suggest future promise in providing an effective solution against CSOM, further tests will be needed before an official treatment package can be released.

It is clear that the impact of CSOM on the developing regions of the world, as well as its current lack of effective treatment options, make it a strong candidate for becoming a true NTD. Ultimately, the global fight against CSOM will require both treatment and prevention strategies, together with efforts to raise awareness of this disease and its health and economic impact on developing countries.

References

1. Orji FT (2013) A Survey of the Burden of Management of Chronic Suppurative Otitis Media in a Developing Country. Ann Med Health Sciences Res 3: 598–601. doi: 10.4103/2141-9248.122126 PMID: 24380016
2. World Health Organization (2004) Chronic suppurative otitis media: Burden of Illness and Management Options. Child and Adolescent Health and Development: Prevention of Blindness and Deafness. Geneva, Switzerland. http://apps.who.int/iris/bitstream/10665/42941/1/9241591587.pdf, accessed September 11, 2014.
3. Kenna M (1988) Etiology and pathogenesis of chronic suppurative otitis media. Arch Otolaryngology—Head Neck Surgery 97 (Suppl. 137): 16–17.

4. Monasta L, Ronfani L, Marchetti F, Montico M, Brumatti LV, et al. (2012) Burden of Disease Caused by Otitis Media: Systematic Review and Global Estimates. PLoS One, 7(4):e36226. doi: 10.1371/journal.pone.0036226 PMID: 22558393

5. Elemraid MA, Brabin BJ, Fraser WD, Harper G, Faragher B, et al. (2010) Characteristics of hearing impairment in Yemeni children with chronic suppurative otitis media: a case-control study. Int J of Ped Otorhinolaryngology, 74: 283–286. doi: 10.1016/j.ijporl.2009.12.004 PMID: 20042241

6. Teele DW, Klein JO, Chase C, Menyuk P, Rossner B, The Greater Boston Otitis Media Study Group (1990) Otitis media in infancy and intellectual ability, school achievement, speech and language at age 7 years. J of Inf Dis 162: 658–694.

7. Murray CJ, Vos T, Lozano R, Naghavi M, Flaxman AD, et. al. (2012) Disability-adjusted life years (DALYs) for 291 diseases and injuries in 21 regions, 1990–2010: a systematic analysis for the Global Burden of Disease Study 2010. Lancet 380:2197–223. doi: 10.1016/S0140-6736(12)61689-4 PMID: 23245608

8. World Health Organization (2014) CSOM: Diseases http://www.who.int/neglected_diseases/diseases/otitis/en/#, Accessed August 16, 2014

9. Yorgancilar E, Yildirim M, Gun R, Bakir S, Tekin R, et al. (2013) Complications of chronic suppurative otitis media: a retrospective review. Eur Arch Otorhinolaryngol 270(1):69–76. doi: 10.1007/s00405-012-1924-8 PMID: 22249835

10. Olatoke F, Ologe FE, Nwawolo CC, Saka MJ (2008) The prevalence of hearing loss among schoolchildren with chronic suppurative otitis media in Nigeria, and its effect on academic performance." Ear, Nose, & Throat J 87: E19.

11. Hanushek E, Peterson PE (2014) Higher Grades, Higher GDP. Hoover Digest 2014 Number 3, 75–78.

12. Srikanth S, Isaac R, Rebekah G, Rupa V (2009) Knowledge, attitudes and practices with respect to risk factors for otitis media in a rural South Indian community Int J Pediatr Otorhinolaryngol 73: 1394–1398. doi: 10.1016/j.ijporl.2009.06.024 PMID: 19640593

13. Li CM, Zhang X, Hoffman HJ, Cotch MF, Themann CL, Wilson MR (2014) Hearing Impairment Associated with Depression in US Adults, National Health and Nutritional Examination Survey 2005–2010. J Am Med Assoc Otolaryngology—Head and Neck Surgery 140:293–302.Paterson JE, Carter S, Wallace Z, Ahmed N, Garett N, Silva PA (2007) Pacific Islands families study: risk factors associated with otitis media with effusion among Pacific 2-year-old children, Int J Pediatr Otorhinolaryngol 71: 1047–1054. PMID: 13640953

14. Cherian T, Bhattacharji S, Brahmadathan KN, Lalitha MK, Raghupathy P, Steinhoff MC (2000) Persistent rhinorrhoea in rural Indian children: prevalence and consequences, J Trop Pediatr 46: 365–367. PMID: 11191150

15. Van der Veen EL, Schilder AG, van Heerbeek N, Zielhuis GA, Rovers MM (2006)Predictors of chronic suppurative otitis media in children. Arch Otolaryngol Head Neck Surg 132: 1115–1118. PMID: 17043261

16. Sophia A, Isaac R, Rebekah G, Brahmadathan K, Rupa V. Risk factors for otitis media among preschool, rural Indian children. Int J Pediatr Otorhinolaryngol 2010; 74: 677–683. doi: 10.1016/j.ijporl.2010.03.023 PMID: 20416956

17. World Health Organization (2009) Effectiveness of Shortened Course ($\leq$ 3 Days) of Antibiotics for Treatment of Acute Otitis Media in Children. Department of Child and Adolescent Health and Development. http://whqlibdoc.who.int/publications/2009/9789241598446_eng.pdf?ua=1, accessed September 11, 2014.

18. Leach AJ, Morris PS, Matthews JD, Chronic Otitis Media Intervention Trial—One (COMIT1) group (2008) Compared to placebo, long-term antibiotics resolve otitis media with effusion (OME) and prevent acute otitis media with perforation (AOMwiP) in a high risk population: a randomized controlled trial. BMC Pediatr; 8:23. doi: 10.1186/1471-2431-8-23 PMID: 18513453

19. Alho OP, Jokinen K, Laitakari K, Palokangas J (1997) Chronic suppurative otitis media and cholesteatoma. Vanishing diseases among Western populations? Clin Otolaryngology 22: 358–361. PMID: 9298613

20. Horlbeck D, Boston M, Balough B, Sierra B, Saenz G, et al. (2009) Humanitarian otologic missions: long-term surgical results. Otolaryngology Head Neck Surg 140: 559–565. doi: 10.1016/j.otohns.2008.12.033 PMID: 19328347

21. Chadha S, Moussy F, Friede MH. (2014) Understanding history, philanthropy and the role of WHO in provision of assistive technologies for hearing loss. Disability and Rehabilitation: Assistive Technology 9: 365–367. doi: 10.3109/17483107.2014.908962 PMID: 24734997

22. Nakatani M (2001) Treatment of chronic suppurative otitis media with Broncasma Berna. Ear Nose & Throat Journal 80: 529.

23. Mackenzie GA, Carapetis JR, Leach AJ, Morris PS (2009) Pneumococcal vaccination and otitis media in Australian Aboriginal infants: comparison of two birth cohorts before and after introduction of vaccination. BMC Pediatr 9:14. doi: 10.1186/1471-2431-9-14 PMID: 19228431

24. Syrjanen RK, Kilpi TM, Kaijalainen TH, Herva EE, Takala AK (2001) Nasopharyngeal carriage of Streptococcus pneumoniae in Finnish children younger than two years old. J Infect Dis 184: 451–459. PMID: 11471103
